# An Integrative Pan-Cancer Analysis Revealing MLN4924 (Pevonedistat) as a Potential Therapeutic Agent Targeting Skp2 in YAP-Driven Cancers

**DOI:** 10.3389/fgene.2022.866702

**Published:** 2022-05-24

**Authors:** Chungen Lan, Bo Ni, Tiansuo Zhao, Zekun Li, Junjin Wang, Ying Ma, Weidong Li, Xiuchao Wang

**Affiliations:** ^1^ Department of Breast Cancer Pathology and Research Laboratory, Tianjin Medical University Cancer Institute and Hospital, National Clinical Research Center of Cancer, Tianjin, China; ^2^ Department of Pancreatic Carcinoma, Tianjin Medical University Cancer Institute and Hospital, National Clinical Research Center of Cancer, Tianjin, China; ^3^ Tianjin’s Clinical Research Center for Cancer, Tianjin Medical University Cancer Institute and Hospital, Tianjin, China; ^4^ Key Laboratory of Cancer Prevention and Therapy, Tianjin, China; ^5^ Key Laboratory of Breast Cancer Prevention and Therapy, Tianjin Medical University, Ministry of Education, Tianjin, China

**Keywords:** YAP, pan-cancer, Skp2, proliferation, drug sensitivity, MLN4924

## Abstract

**Background:** YAP, coded by *YAP1* gene, is critical in the Hippo pathway. It has been reported to be involved in the tumorigenesis and progression of several cancers. However, its roles on tumor cell proliferation in diverse cancers remain to be elucidated. And there is currently no clinically feasible drug that can directly target YAP in cancers. This research aimed to explore the regulatory mechanism of YAP in promoting tumor proliferation of multiple cancers, in order to find new strategies for inhibiting the overgrowth of YAP-driven cancers.

**Methods:** We investigated the expression pattern of *YAP1* in pan-cancer across numerous databases and our cohorts. First, univariate Cox regression analysis and survival analysis were used to evaluate the effect of *YAP1* on the prognosis of cancer patients. Second, TIMER was used to explore the relationship between *YAP1* expression and tumor cell proliferation. Third, functional and pathway enrichment was performed to search for targets of YAP involved in cell cycle in cancers. At last, GDSC and CCLE datasets were used to assess the correlation between *SKP2* expression and MLN4924 IC50 values.

**Results:** Differential expression analysis of multiple databases and qPCR validation showed that *YAP1* was generally overexpressed in pan-cancers. Survival analysis revealed that *YAP1* over-expression was significantly related to poor prognosis of patients with PAAD. The expression level of *YAP1* was positively correlated with the proliferation in varieties of tumors. Further, *SKP2* was confirmed as a target of YAP in promoting tumor cell proliferation. In addition, *SKP2* expression was negatively correlated with MLN4924 IC50 values in almost all cancer types.

**Conclusion:** *YAP1* is frequently overexpressed in human cancers. YAP promoted tumor cell proliferation by up-regulating *SKP2* expression in multiple cancers. The comprehensive pan-cancer analysis suggested that inhibition of Skp2 with MLN4924 might be an effective therapeutic strategy for attenuating tumor cell proliferation in YAP-driven cancers.

## Introduction

Cancer arises from the overgrowth of cancer cells, which activates mechanisms that promote cell growth and cancer progression (Hanahan and Weinberg, 2011). A number of studies have proved the essential role of cell signaling pathways in the occurrence and development of cancer (Dandawate et al., 2016). The Hippo pathway, whose constituents are highly conservative in evolution, plays an integral role in tissue repair and controlling the size of organs by regulating cell proliferation (Zhao et al., 2008; Zhao et al., 2011; Lee et al., 2014; Zanconato et al., 2015). Yes-associated protein (YAP) is a transcriptional co-activator and the main nuclear effector of the Hippo pathway. It has been described to function as a critical oncogene by promoting cancer initiation and progression, but dispensable for normal homeostasis in several adult organs (Zanconato et al., 2016). The stark contrast between the insignificance of YAP inactivation for normal organ function and their absolute requirement for cancer development in the same organ is attractive, highlighting the possibility that targeting YAP may show a large therapeutic window.

More than fifty drugs have been shown to inhibit YAP activity (Juan and Hong, 2016), however none other than verteporfin acts directly on YAP. Verteporfin, a YAP inhibitor, is found to be effective to inhibit liver overgrowth due to YAP overexpression (Liu-Chittenden et al., 2012). However, the use of verteporfin may cause adverse effects on patients due to its inhibition of autophagy, high phototoxicity, and induction of oligomerization (Wang et al., 2016; Gibault et al., 2017). These caveats, along with its poor pharmacokinetics, suggest that this compound may not translate well to the clinic. Therefore, novel therapeutic strategies are urgently needed for the treatment of YAP-driven cancers.

Skp2 was originally defined as S-phase kinase-associated protein 2, and its expression level changes periodically as the cell cycle progresses (Bashir and Pagano, 2004; Cai et al., 2020). It was reported that aberrant Skp2 expression and dysregulation of its activity were closely associated with various human cancers (Hao and Huang, 2015). Skp2 was a well-characterized component of Skp2-SCF E3 ligase complex composed of Skp1, Skp2, Cullin 1 (Cul-1), and Roc1/Rbx1 (Cai et al., 2020). Previous studies have shown that neddylation of Cul-1 is critical for the functional activity of the Skp2-SCF complex (Liu et al., 2002). Pevonedistat (MLN4924) is a synthesized small molecule inhibitor of NEDD-activating enzyme (Soucy et al., 2009a). MLN4924 could specifically inhibit NEDD8-activating enzyme and compromise Cul-1 neddylation, consequently causing deconstruction and deactivation of Skp2-SCF complex (Chen and Tweddle, 2012).

In this study, we firstly investigated the expression pattern and prognostic value of *YAP1* in pan-cancer analysis using multiple databases and Kaplan-Meier plotter. Then, we explored the clinical significance of YAP expression levels in pancreatic adenocarcinoma (PAAD). We found that the expression of *YAP1* in PAAD was significantly stronger than that of adjacent normal tissues. And our results indicated that the YAP expression level in PAAD tissue could be used to predict the prognosis of patients and that the YAP expression in PAAD was positively correlated with histological grade and tumor proliferation. Further, we tried to collect the statistical power of the pan-cancer technique on large numbers of clinical samples to reveal the relationship between *YAP1* and *MKI67* using TIMER. We found that *YAP1* expression positively correlates with tumor proliferation in almost all cancer types. Of note, we confirmed that *SKP2* was a target of YAP and involved in the proliferation of tumor cells in the cell cycle process of pan-cancer by bioinformatic analysis. Specifically, considering the current situation that there were no clinically viable drugs that directly target YAP in cancer, MLN4924, as a drug that could inhibit the activity of Skp2, might be a potential treatment agent for attenuating the proliferation of tumor cells in YAP-driven cancers.

## Materials and Methods

### TIMER Database Analysis

TIMER (Li et al., 2017) is a comprehensive resource that can be used to systematically analyze the immune infiltration of different cancer types (https://cistrome.shinyapps.io/timer/). Its differential expression (DiffExp) module was used to study the differential expression of any gene of interest in the cancer genome atlas (TCGA) between the tumor and adjacent normal tissues. The distribution of gene expression levels was shown using box plots, and the statistical significance of differential expression was evaluated using Wilcoxon’s test. Its correlation module was used to draw a scatter plot of expression between a pair of user-defined genes in a given cancer type, as well as Spearman’s rho value and estimated statistical significance.

### Prognostic Analysis

Forest plots and Kaplan-Meier curves were used to examine the relationship between *YAP1* expression and the survival rates of 33 types of cancer. A univariate Cox regression survival analysis was used to calculate the hazard ratio (HR) and 95% confidence interval in a given cancer type. The prognostic significance of *YAP1* expression was evaluated with overall survival (OS) and disease-free survival (DFS).

### GEPIA Database Analysis

GEPIA (http://gepia.cancer-pku.cn) (Tang et al., 2017) is a website based on TCGA and Genotype-Tissue Expression (GTEx) projects cancer data mining. We analyzed the differential expression of *YAP1* in PAAD and matched TCGA normal and GTEx data through GEPIA. The correlation between *YAP1* expression and advanced pathological stage in PAAD was analyzed by GEPIA. Patient survival analysis based on the expression of *YAP1* in PAAD (OS and DFS) and Brain Lower Grade Glioma (LGG) (OS) were conducted by GEPIA.

### DEGs Analysis

All GSE datasets used in this article were obtained from Gene Expression Omnibus (GEO) database (https://www.ncbi.nlm.nih.gov/geo/) (Clough and Barrett, 2016). Limma package of R software was used to study the differential expression of mRNAs in GSE66949, GSE49406, and GSE163110. GSE32597, GSE35004, and GSE92335 were screened using the GEO2R tool to identify genes that were differentially expressed. A *p* value < 0.05 and **|**Fold change**|** > 1.5 were used as the threshold to identify differential expression genes (DEGs). FunRich software was used to find overlapping DEGs between GSE66949 and GSE32597 datasets. The overlapping DEGs were further analyzed to identify the most commonly regulated genes across datasets. The volcano plots and heat map for hierarchical clustering of DEGs were drawn by R language.

### Metascape Analysis

Metascape is a powerful and efficient tool designed to provide users with comprehensive gene list annotation and analysis resources (Zhou et al., 2019). Pathway and process enrichment analyses of the overlapping DEGs between GSE66949 and GSE32597 cohorts were performed with Metascape using default parameters. The Molecular Complex Detection tool (MCODE) could screen and identify the most significant module in the PPI network.

### Gene Ontology and Kyoto Encyclopedia of Genes and Genomes Pathway Enrichment Analysis of DEGs

The ClusterProfiler package in R v4.0.3 was used to determine the functions of DEGs identified in GO (Gene Ontology Consortium, 2015) and KEGG (Kanehisa et al., 2016) pathway analysis, respectively.

### *SKP2* Expression Analysis by CCLE Dataset

The *SKP2* expression matrix of 32 tumor cell lines was obtained from the CCLE dataset (https://portals.broadinstitute.org/ccle/about). The analysis was constructed by the R v4.0.3 software package ggplot2 (v3.3.3).

### Spearman Correlation Analysis of *SKP2* Gene Expression and MLN4924 IC50 Score

For the TCGA database, we downloaded tumor RNA-seq (FPKM) from the Genomic Data Commons (GDC). We converted PFKM data to TPM and normalized the data log2 (TPM+1), while keeping samples with clinical information recorded. We predicted the chemotherapeutic response of each sample based on the largest public pharmacogenomics database the Genomics of Drug Sensitivity in Cancer (GDSC), https://www.cancerrxgene.org. The prediction process was implemented by R package “pRRophetic,” where the half-maximum inhibitory concentration (IC50) of the sample was estimated through ridge regression and the prediction accuracy. All parameters were set by default values, eliminating the batch effect of combat and tissue types of all cancers, and repeated gene expression was summarized as the average value. All the above analysis methods and R package were implemented by R foundation for statistical computing (2020) version 4.0.3.

### *YAP1* and *SKP2* Expression Pattern in Human Pan-Cancer

The *YAP1* expression profile in pan-cancer was analyzed using TIMER database. The gene expression levels were presented as log2 TPM values.

By combining normal tissue data from the GTEx database with data from TCGA, the dysregulation of *YAP1* and *SKP2* expression between various cancers and normal tissues was studied. RNA sequencing data for patients with 33 types of cancers, including adrenocortical carcinoma (ACC), bladder urothelial carcinoma (BLCA), breast invasive carcinoma (BRCA), cervical squamous cell carcinoma and endocervical adenocarcinoma (CESC), cholangiocarcinoma (CHOL), colon adenocarcinoma (COAD), lymphoid neoplasm diffuse large B cell lymphoma (DLBC), esophageal carcinoma (ESCA), glioblastoma (GBM), LGG, head and neck squamous cell carcinoma (HNSC), kidney chromophobe (KICH), kidney renal clear cell carcinoma (KIRC), kidney renal papillary cell carcinoma (KIRP), acute myeloid leukemia (LAML), liver hepatocellular carcinoma (LIHC), lung adenocarcinoma (LUAD), lung squamous cell carcinoma (LUSC), mesothelioma (MESO), ovarian serous cystadenocarcinoma (OV), PAAD, pheochromocytoma and paraganglioma (PCPG), prostate adenocarcinoma (PRAD), rectum adenocarcinoma (READ), sarcoma (SARC), skin cutaneous melanoma (SKCM), stomach adenocarcinoma (STAD), testicular germ cell tumors (TGCT), thyroid carcinoma (THCA), thymoma (THYM), uterine corpus endometrial carcinoma (UCEC), uterine carcinosarcoma (UCS), and uveal melanoma (UVM), were obtained from the TCGA database. All expression data were normalized *via* log2 conversion.

### Immunohistochemistry

Paraffin-embedded pancreatic cancer tissue microarray containing 130 PAAD patient samples was approved by the Ethics Committee of Tianjin Medical University Cancer Institute and Hospital, China. All specimens were handled and made anonymous according to ethical and legal standards. All patients included in this study had no history of other malignancies and none of them had received chemotherapy, radiotherapy, and other treatment prior to surgery. Histopathology review and diagnosis confirmation were performed by three independent pathologists. Histological grade was classified into highly differentiated (G1), moderately differentiated (G2), and poorly differentiated (G3). Immunohistochemistry for YAP (#12395, 1:350, CST, United States) and Ki-67 (ab92742, 1:500, Abcam, United Kingdom) were performed using standard techniques. Two pathologists evaluated staining. Nuclear staining for YAP and Ki67 was considered positive. The immunoreactivity of YAP was scored based on the staining intensity and the percentage of cells stained positively. Sections for Ki67 were classified as follows: low (0%–25% of stained cells) and high (26%–100% of stained cells).

### Tumor Tissue Collection

Pancreatic cancer, glioma, ovarian cancer, and colorectal cancer tissues and adjacent normal tissues were collected from Tianjin Medical University Cancer Institute and Hospital, China. They were stored immediately in liquid nitrogen and kept at −80°C. None of the patients had received neoadjuvant chemotherapy or preoperative radiation therapy. The study was approved by the Ethics Committee of Tianjin Medical University Cancer Institute and Hospital and conducted following the Declaration of Helsinki.

### RNA Extraction and RT-qPCR

Total RNA was isolated from cells using Trizol reagent (Invitrogen, Carlsbad, CA, United States ) according to the manufacturer’s instructions. cDNA was generated by the RTase M-MLV (Takara, Shiga-ken, Japan) as described in the manufacturer’s protocol. Quantification of *YAP1* mRNA level was done by RT-qPCR using SYBR Green PCR Master Mix (TaKaRa, Shiga-ken, Japan), and the expression of GAPDH was used as the internal control. The following primers were used to amplify target genes: *GAPDH*, 5′-CAG​GAG​GCA​TTG​CTG​ATG​AT-3′, 5′-GAA​GGC​TGG​GGC​TCA​TTT-3’; *YAP1*, 5′-AGT​GGA​CTA​AGC​ATG​AGC​AG-3′, 5′-TGT​TCA​TTC​CAT​CTC​CTT​CC-3'. Fold changes were calculated using the ΔΔCt method in Microsoft Excel.

### Statistical Analysis

Correlations between two variables were carried out by Spearman’s Rank-Correlation test or Pearson correlation analysis. The Kaplan-Meier method and log-rank test were applied to evaluate the relationship of different gene expression levels with OS, DFS, and RFS in certain tumor types. Univariate and multivariate Cox proportional hazards analyses were performed to assess the potential impact of YAP expression on OS and RFS in PAAD. Analyses were performed using SPSS 22.0 statistical analysis software or Graphpad 8.0 or R 4.0.3 software packages. Unpaired Student’s t test was used for two-group comparison. A two-sided *p* < 0.05 was considered statistically significant.

## Result

### Pan-Cancer Expression Landscape of *YAP1*

**FIGURE 1:**
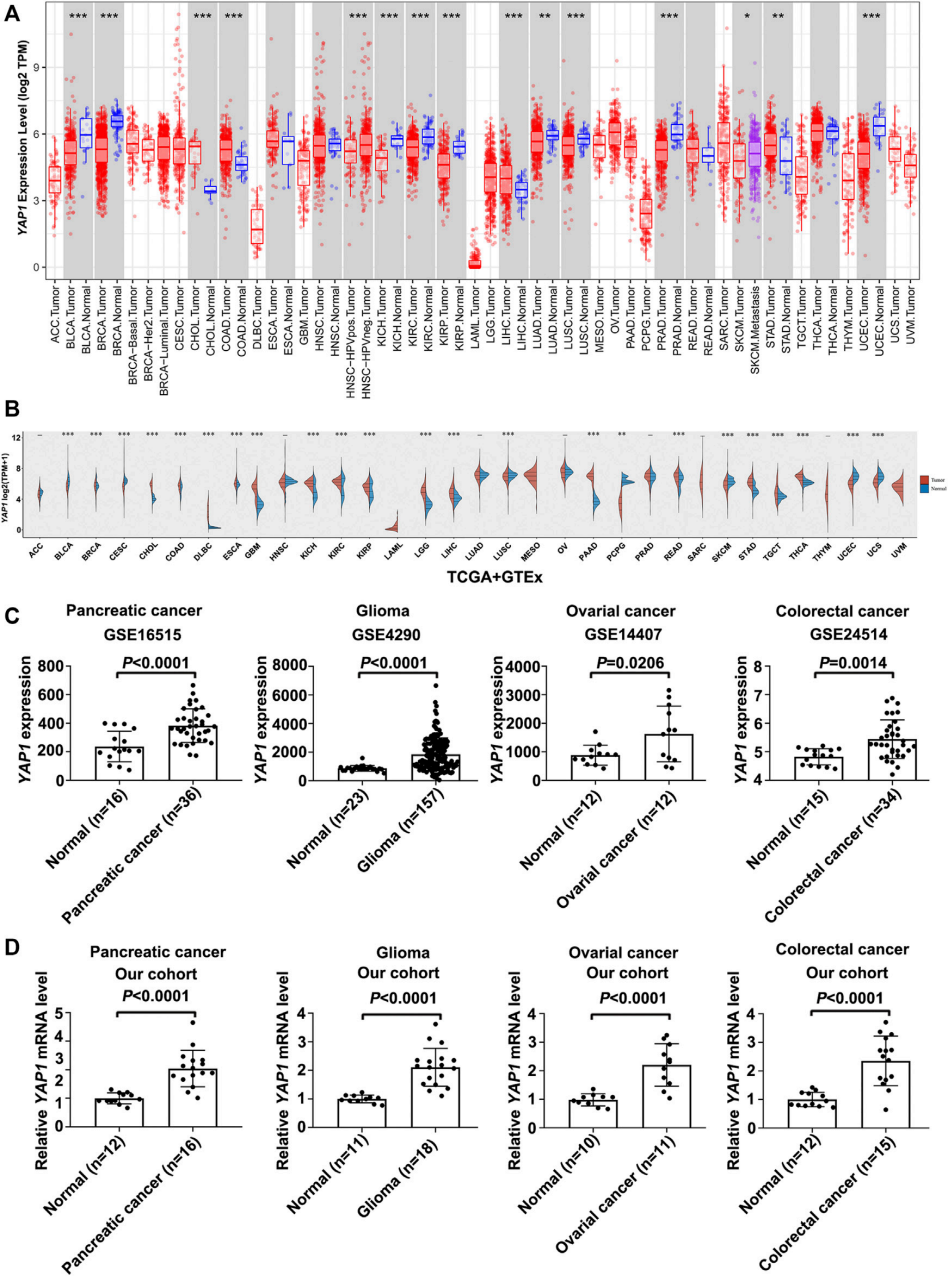
The expression analysis of *YAP1* in Pan-cancer. **(A)** The *YAP1* expression in tumors and adjacent normal tissues in the pan-cancer data of the TCGA cohort. **(B)** The expression level of *YAP1* in different cancer types from TCGA and GTEx data. The red spindle represents tumor tissue, and the blue spindle represents normal tissue. X axis, different tissue type. Y axis, *YAP1* expression. *p < 0.05, **p < 0.01, and ***p < 0.001. **(C,D)** The *YAP1* mRNA expression in cancer tissues of four cancer types, including pancreatic cancer, glioma, ovarian cancer, and colorectal cancer were significantly overexpressed compared with normal tissues in GSE16515, GSE4290, GSE14407, and GSE24514 database **(C)** and in our cohorts **(D)**. Data were presented as mean ± SD and *p*-value was generated by Student’s t-test.

According to the results from the TIMER database, *YAP1* exhibited inconsistent mRNA expression in 33 types of common cancers. Compared with adjacent normal tissues, *YAP1* expression in cancer was significantly higher in CHOL, COAD, LIHC, STAD (Figure 1A). We also compared *YAP1* expression using the data from TCGA and GTEx databases. Compared with normal tissues, the upregulated *YAP1* mRNA expression was observed consistently in tumor tissues in CHOL, COAD, LIHC, STAD, and moreover in DLBC, ESCA, GBM, KICH, KIRC, KIRP, LGG, PAAD, READ, STAD, TGCT, and THCA (Figure 1B). Besides, the expressions of *YAP1* in cancer and normal tissues of four cancer types, including pancreatic cancer, glioma, ovarian cancer, and colorectal cancer were verified by GSE16515, GSE4290, GSE14407, and GSE24514 (Figure 1C). We found that *YAP1* was highly expressed in cancer tissues, as compared to adjacent normal tissues. The expression of *YAP1* was further examined by qPCR in pancreatic cancer, glioma, ovarian cancer, and colorectal cancer tissue samples and the corresponding normal tissue samples. As shown in Figure 1D, these results consistently showed that the expression of *YAP1* in pancreatic cancer, glioma, ovarian cancer, and colorectal cancer were significantly higher than the corresponding normal tissues. Data above indicated that *YAP1* might play a role as an oncogene in the development of varieties of tumors.

### Pan-Cancer Analysis of the Prognostic Value of *YAP1*

**FIGURE 2:**
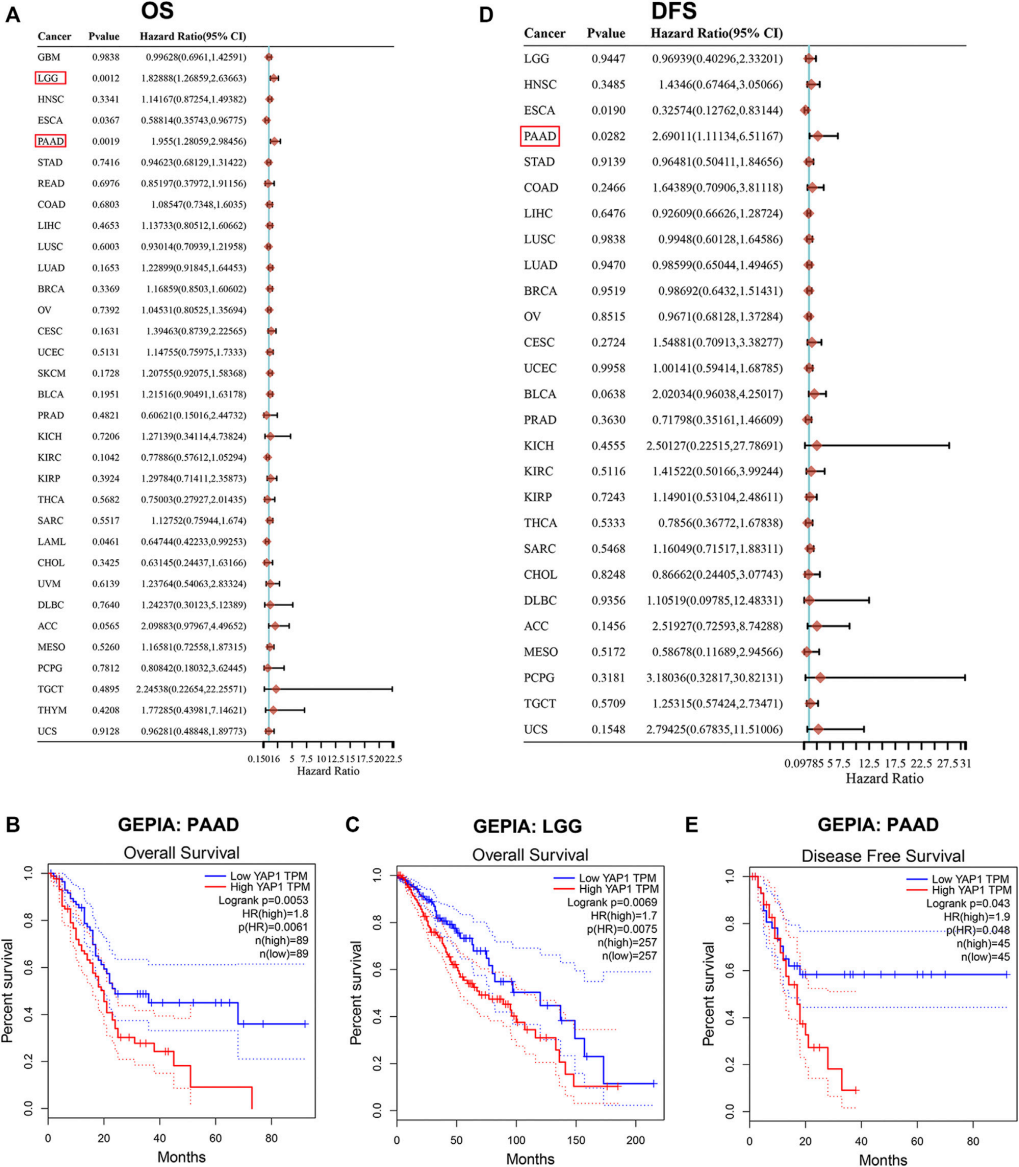
Prognosis value of *YAP1* in TCGA pan-cancer. **(A)** Forrest plots of results from the univariate survival analysis in pan-cancer for OS. Diamonds indicate the hazard ratios (HRs) from the univariate overall-survival analysis, and HR < 1 represents a favorable factor, while HR > 1 represents an adverse factor. The 95% confidence interval (95% CI) is shown as the horizontal line across the diamond. **(B-C)** Kaplan-Meier OS analysis of *YAP1* in PAAD and LGG by GEPIA. **(D)** Forrest plots of results from the univariate survival analysis in pan-cancer for DFS. **(E)** Kaplan-Meier DFS analysis of *YAP1* in PAAD by GEPIA.

Next, we studied the prognostic value of *YAP1* by pan-cancer analysis in different databases. Firstly, we utilized univariate Cox proportional hazard regression models to analyze the association between *YAP1* expression in pan-cancer and OS or DFS. Cox regression analysis results from 33 types of cancer suggested that *YAP1* expression positively correlated with poor OS in patients with PAAD and LGG with significance (Figure 2A). Kaplan-Meier survival curves indeed indicated that the upregulated *YAP1* expression was significantly associated with poor OS in patients with PAAD and LGG (Figures 2B,C). Then, we examined the relationship between *YAP1* expression and the DFS of patients with cancer. The *YAP1* expression affected DFS with statistical significance in patients with PAAD (Figure 2D). Kaplan-Meier analysis also showed that the increased *YAP1* expression corresponded to the poor DFS in patients with PAAD (Figure 2E).

### *YAP1* Expression Directly Correlates With PAAD Malignant Proliferation

**FIGURE 3:**
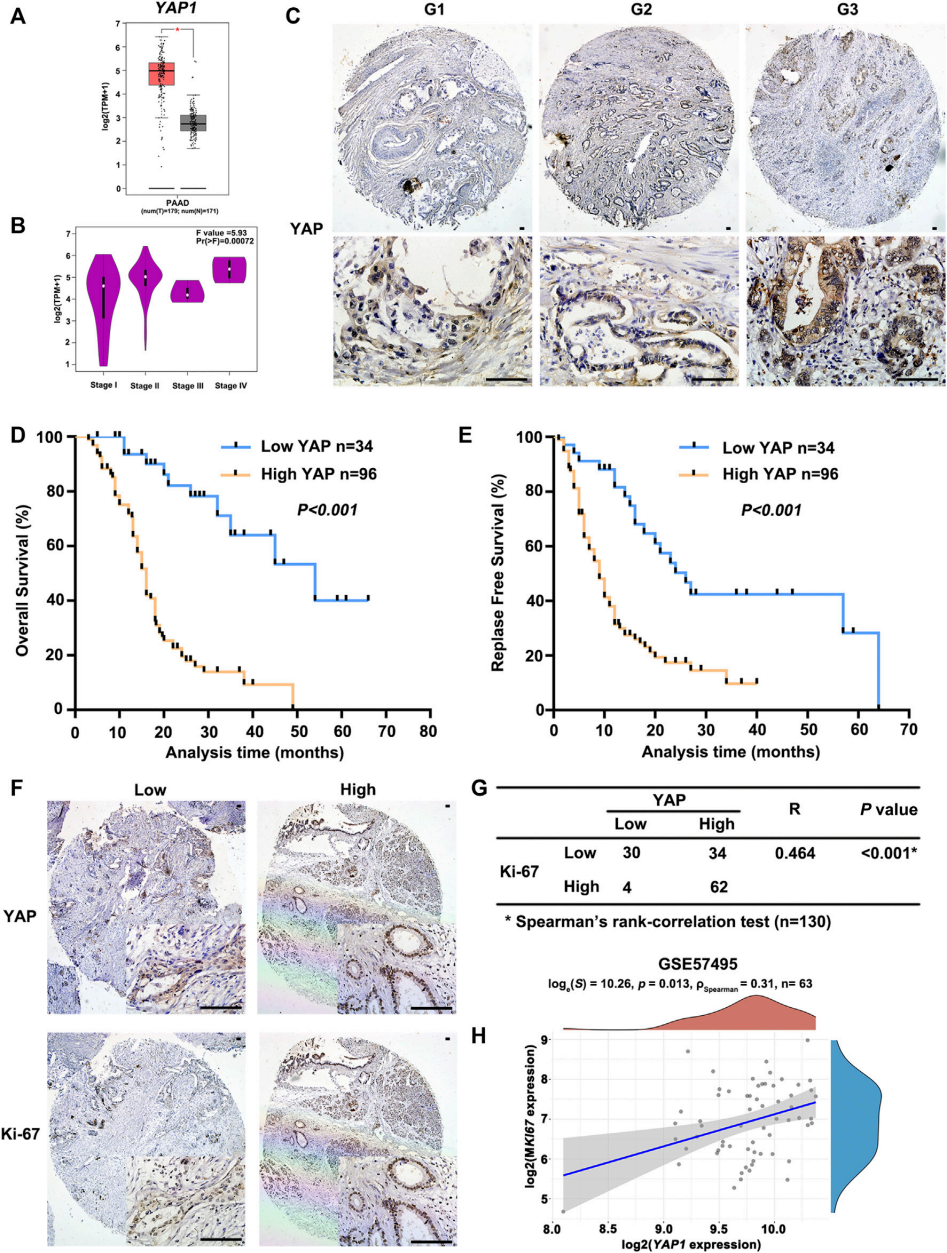
The positive correlation between the expression of *YAP1* and malignant potential of PAAD. **(A)** The transcriptional profile of *YAP1* was analyzed in PAAD tissue samples (T) and normal tissue samples (N) obtained from PAAD datasets in TCGA analyzed by GEPIA. **(B)** Significant differences in *YAP1* expression in different pathological stages were analyzed by GEPIA. **(C)** Representative images of YAP immunostaining in PAAD with different histological grades. G1, highly differentiated; G2, moderately differentiated; G3, poorly differentiated. Scale bar: 100 μm. **(D)** Kaplan-Meier curves of overall survival in PAAD patients with YAP expression. According to the immunostaining of YAP, the patients (*n* = 130) were divided into two groups with low or high expression. *p* value was calculated by the log-rank test. **(E)** Kaplan-Meier curves of relapse-free survival in PAAD patients with YAP expression. *p* value was calculated by the log-rank test. **(F)** YAP and Ki67 expression in serial sections of human PAAD tissue microarray by immunostaining. Scale bar: 100 μm. **(G)** Correlation analysis of immunostaining results of YAP and Ki67 expression in PAAD tissue microarray. **(H)** Spearman correlation analysis of *YAP1* expression and *MKI67* expression, the value represents the correlation *p* value.

As shown in Figure 3A, *YAP1* expression was significantly higher in PAAD tissues compared to normal tissues. In addition, the increased expression of *YAP1* was notably associated with the advanced pathological stage (Figure 3B). To learn more about the role of YAP in PAAD, we examined the expression of YAP in a tissue microarray composed of 130 human PAAD samples through immunohistochemistry assays. YAP staining was detected in all PAAD tissues and the expression of nuclear YAP was positively correlated with the histological grade of PAAD (Supplementary Table S1; Figure 3C). Importantly, Kaplan-Meier analysis showed that PAAD patients with high YAP protein expression had significantly worse OS and RFS than patients with low YAP expression (Figures 3D,E). Univariate and multivariate cox proportional hazards analysis also indicated that upregulation of YAP expression is an independent risk factor for PAAD patients (Supplementary Table S2). In addition, to assess the role YAP played in PAAD proliferation, we used Ki67 as a proliferation indicator and found a positive correlation between YAP and Ki67 expression in PAAD samples (Figures 3F,G). In the GSE57495 cohort, the *YAP1* and *MKI67* mRNA levels were also positively correlated in PAAD (Figure 3H). Taken together, these results indicated that YAP played an extremely important role in PAAD cell proliferation.

### *YAP1* Expression Positively Correlates With the Proliferation of Pan-Cancer

**FIGURE 4:**
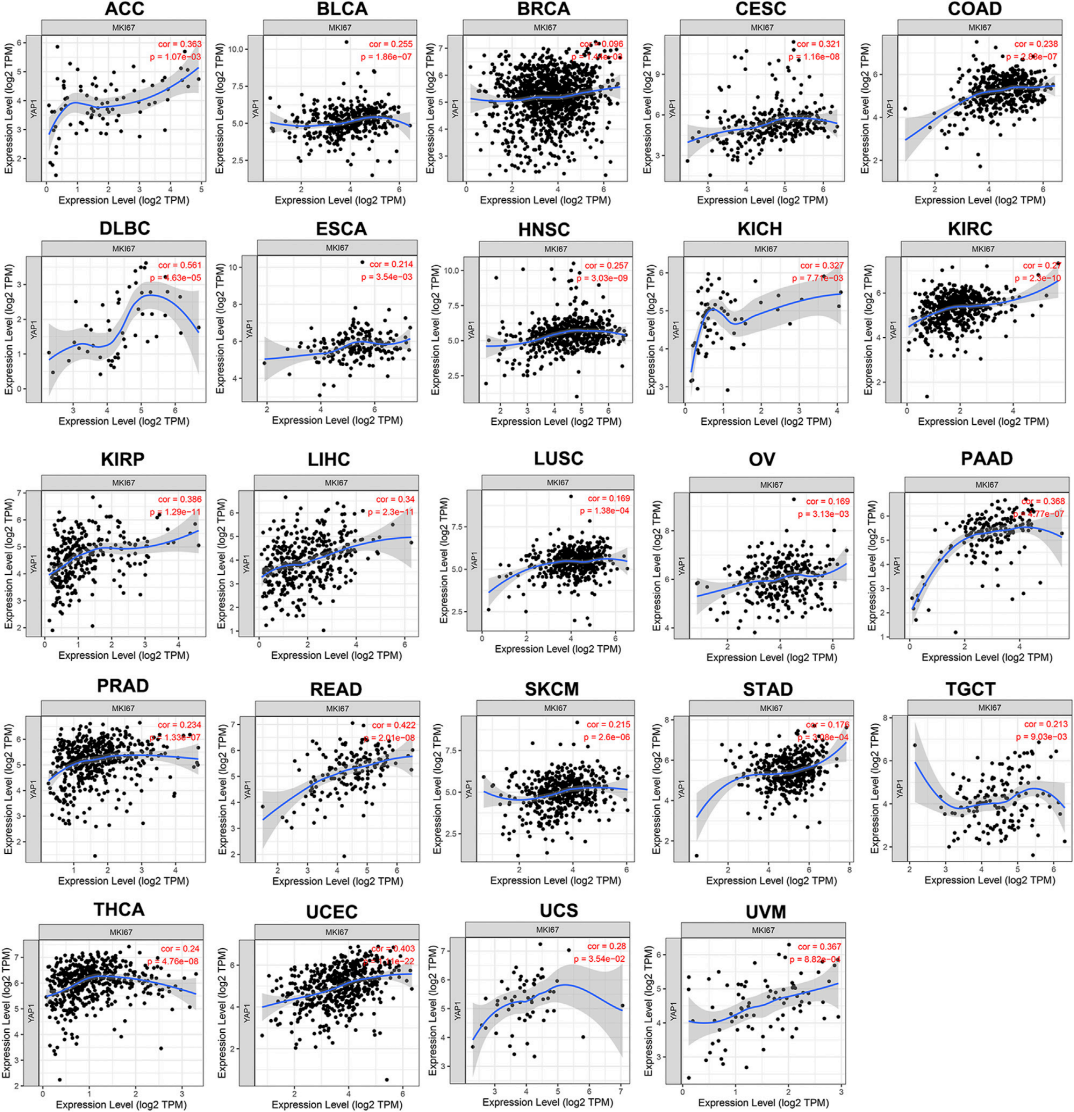
The positive correlation between *YAP1* and *MKI67* expression analyzed by TIMER in pan-cancer.

TIMER results noted that *YAP1* expression was positively correlated with *MKI67* in 24 types of tumors, including ACC, BLCA, BRCA, CESC, COAD, DLBC, ESCA, HNSC, KICH, KIRC, KIRP, LIHC, LUSC, OV, PAAD, PRAD, READ, SKCM, STAD, TGCT, THCA, UCEC, UCS, and UVM (Figure 4). Collectively, these findings indicated that *YAP1* expression positively correlated with the proliferation of multiple tumors.

### *SKP2* Regulated by YAP Participates in the Cell Cycle Process of Multiple Tumors

**FIGURE 5:**
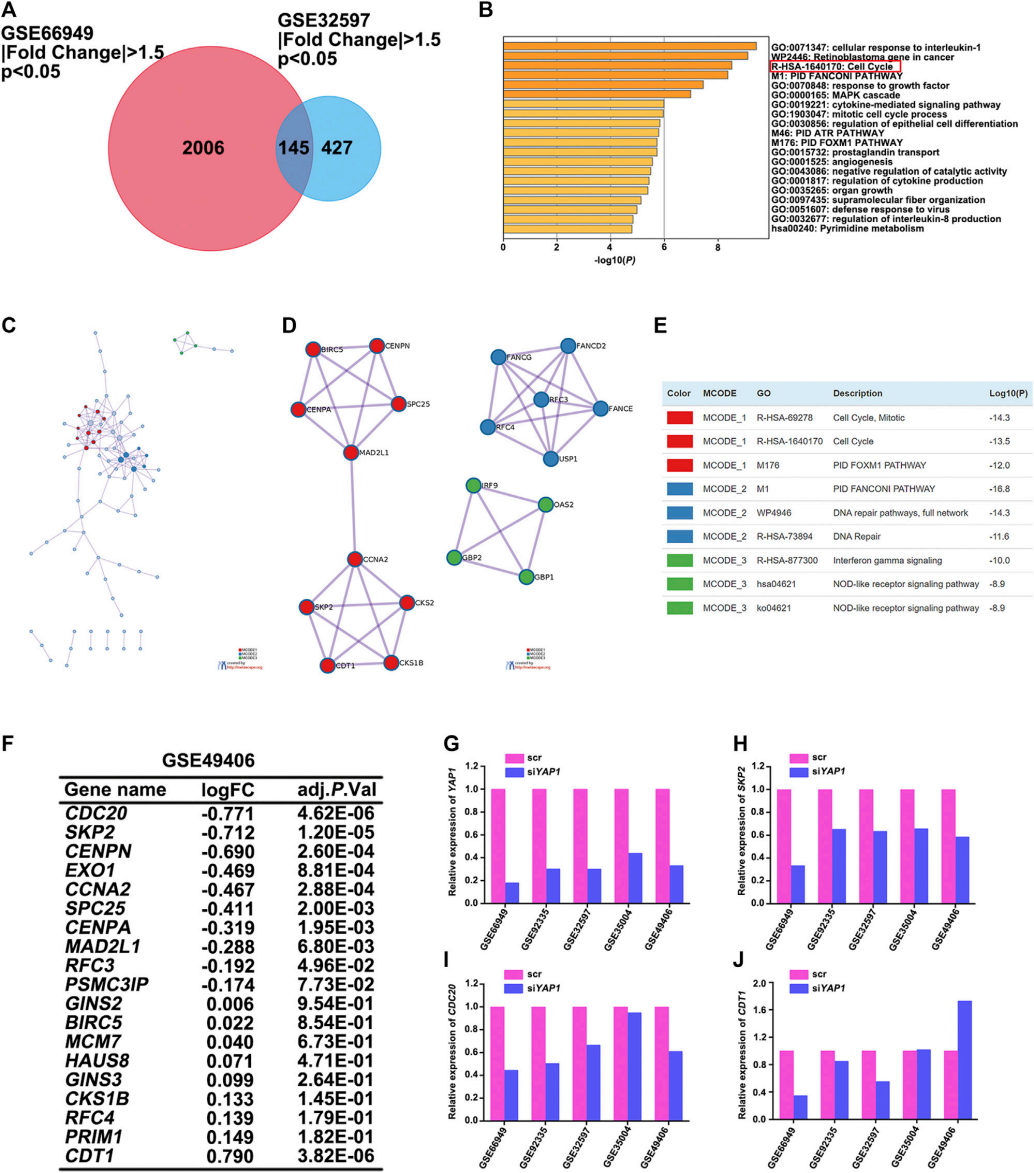
*SKP2* regulated by YAP participated in the cell cycle process of multiple tumors. **(A)** Identification of DEGs in mRNA expression profiling datasets GSE66949 and GSE32597 with an adjusted *p* < 0.05, **|**Fold Change**|** >1.5 as the cut-off criteria. Venn diagram displaying the overlap between DEGs from GSE66949 and GSE32597 datasets. **(B)** Metascape functional enrichment analysis of the overlapping DEGs between GSE66949 and GSE32597 cohorts. **(C)** A protein-protein interaction (PPI) network of the overlapping DEGs was constructed in Metascape. **(D)** Modules selected from PPI network using MCODE. **(E)** The description of the top three MCODE components. **(F)** Analysis of the expression changes of overlapping DEGs participated in the cell cycle process between GSE66949 and GSE32597 in GSE49406. **(G)** The relative expression level of *YAP1* between control and si*YAP1* group in 5 GEO datasets. **(H)** The relative expression level of *SKP2* between control and si*YAP1* group in 5 GEO datasets. **(I)** The relative expression level of *CDC20* between control and si*YAP1* group in 5 GEO datasets. **(J)** The relative expression level of *CDT1* between control and si*YAP1* group in 5 GEO datasets.

Although the Hippo-YAP signaling pathway has been extensively studied over the past years and it has been reported that cyclins like cyclin D1 and E2F1 are involved in YAP function (Nicolay et al., 2011; Mizuno et al., 2012), the full range of YAP target genes that regulate cell cycle processes has not yet been identified. Therefore, we performed the analysis of differential expression of mRNAs between the control and si*YAP1* groups. Limma package of R software was used to study the differential expression of mRNAs in GSE66949. GSE32597 was screened using the GEO2R tool to identify genes that were differentially expressed. A total of 2,151 and 572 genes were identified as the DEGs from their respective datasets (Figure 5A). Next, FunRich software was used to identify overlapping DEGs between GSE66949 and GSE32597. We found 145 overlapping genes (Figure 5A), which were used for functional and pathway enrichment analysis using the online gene annotation tool Metascape (Zhou et al., 2019). As demonstrated in Figure 5B, the enrichment results mainly presented its involvement of cellular response to interleukin-1, retinoblastoma gene in cancer, cell cycle, etc. The protein-protein interaction (PPI) topology analysis of the overlapping DEGs was performed in order to systemically learn the functions of YAP target genes. The PPI network and Molecular Complex Detection (MCODE) components identified in 145 aforementioned DEGs were shown in Figures 5C–E. The three most significant MCODE components were extracted from the PPI network. After pathway and process enrichment analysis was independently applied to each MCODE component, the results showed that its biological function was mainly related to the cell cycle, DNA repair, and interferon-gamma signaling. On the basis of the enrichment analysis, YAP was determined to play an important role in the cell cycle of multiple tumors.

To more clearly figure out the target genes that were regulated by YAP with its participation in the cell cycle process of multiple tumors, we analyzed GSE49406 using the Limma package of R software in *YAP1*-knockdown and control HEK293 cells. The expression changes of overlapping DEGs that participated in the cell cycle process in GSE66949 and GSE32597 were assessed. As shown in Figure 5F, the expressions of *CDC20*, *SKP2*, and *CDT1* were significantly changed. The changes in expression patterns of *SKP2*, *CDC20*, and *CDT1* were further assessed based on the inhibition of *YAP1* in GSE66949, GSE92335, GSE32597, GSE35004, and GSE49406 (Figures 5G–J). Details of the *YAP1* inhibition datasets from the GEO database were shown in Table 1. As the bar charts showed, *SKP2* and *CDC20*, especially *SKP2*, exhibited the same expression trends with the inhibition of *YAP1* in all 5 datasets (Figures 5H–I). But *CDT1* exhibited an inconsistent trend with the expression pattern of *YAP1* in the GSE datasets above (Figure 5J). It is worth noting that only *SKP2* exhibited the same expression pattern with statistical significance based on the inhibition of *YAP1* in all 5 datasets (Table 2). Moreover, stable cell lines with downregulation of YAP in four cancer types, including pancreatic cancer, glioma, ovarian cancer, and colorectal cancer, were established. Knockdown of YAP expression correlated well with decreased *SKP2* protein and mRNA levels (Supplementary Figure S1). To sum up, *SKP2,* as the target gene regulated by YAP, participates in the cell cycle process of multiple tumors.

**TABLE 1:** Details of the YAP1 inhibition datasets from the GEO database.

GSE	Cell type	Tumor type	Upregulated DEGs	Downregulated DEGs	Platform	Sample size
GSE66949	SCC2	OSCC	1,102	1,049	GPL17889	3 sicontrol VS. 3 siYAP1
GSE32597	SK-Hep1	LIHC	382	190	GPL10558	3 sicontrol VS. 3 siYAP1
GSE92335	HCT116	COAD	200	28	GPL570	3 sicontrol VS. 3 siYAP1
GSE35004	Hep3B	LIHC	2	41	GPL6244	3 sicontrol VS. 3 siYAP1
GSE49406	HEK293	—	846	564	GPL10558	3 WT VS. 3 siYAP1

**TABLE 2:** The expression change of *YAP1*, *SKP2*, *CDC20,* and *CDT1* based on the inhibition of *YAP1* in 5 GEO datasets.

Gene name	GSE66949		GSE92335		GSE32597		GSE35004		GSE49406	
	logFC	*p* value	logFC	*p* value	logFC	*p* value	logFC	*p* value	logFC	*p* value
*YAP1*	−2.461	7.14492E-09	−1.720	4.98E-09	−1.730	8.25E-08	−1.184	6.83E-08	−1.587	2.18896E-11
*SKP2*	−1.579	4.87467E-08	−0.615	0.0000727	−0.653	0.0000594	−0.605	0.00000348	−0.712	8.16529E-07
*CDC20*	−1.166	4.06017E-07	−0.988	0.00000114	−0.588	0.0000735	−0.075	0.249	−0.771	2.13511E-07
*CDT1*	−1.521	1.80194E-06	−0.232	0.0605	−0.851	0.000315	0.026	0.691	0.790	1.66801E-07

### *SKP2,* Instead of *CDC20*, Positively Correlates With *YAP1* Expression in Pan-Cancer

**FIGURE 6:**
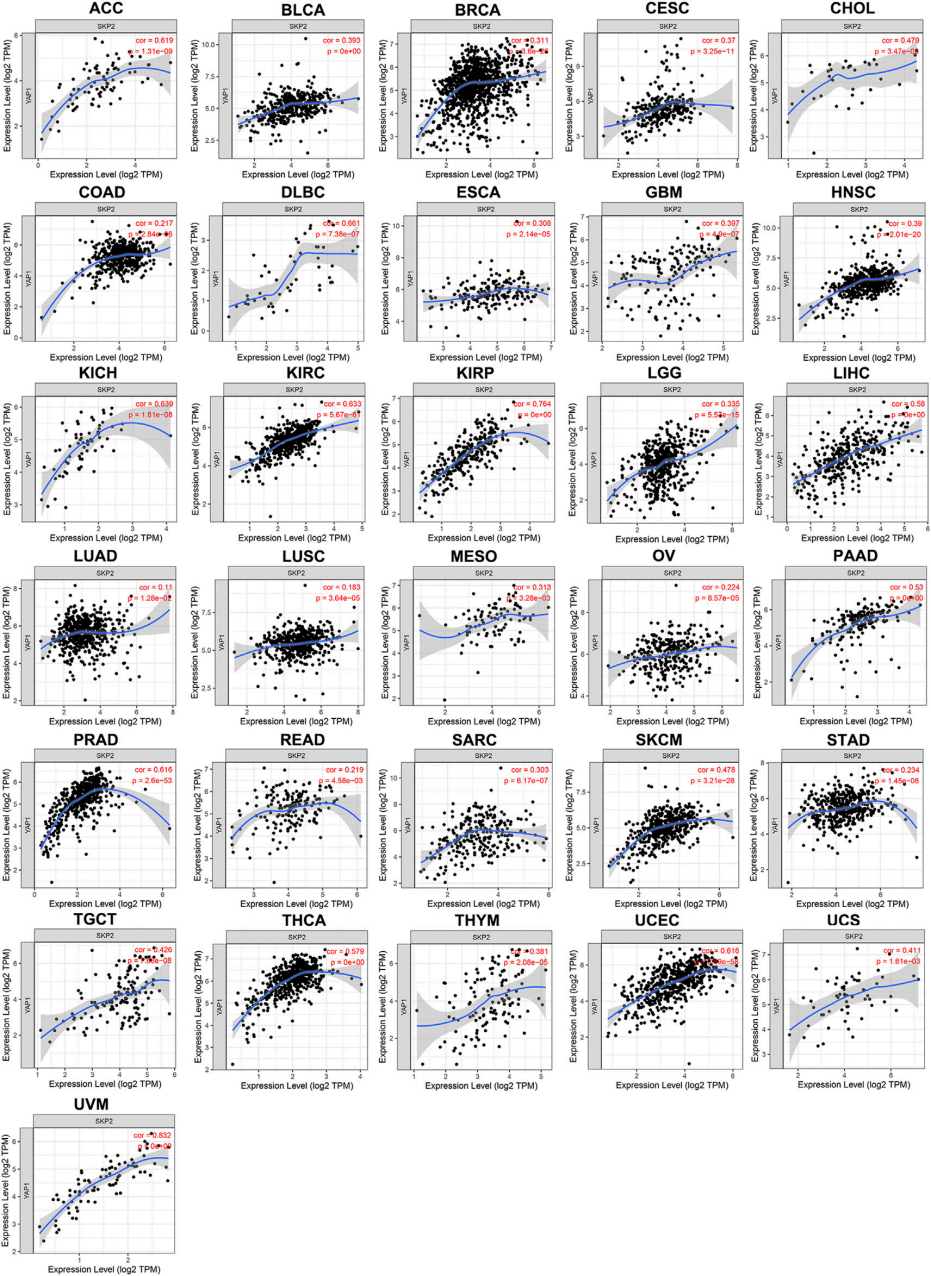
The positive correlation between *YAP1* and *SKP2* expression analyzed by TIMER in pan-cancer.

TIMER results noted that *SKP2* expression was positively correlated with *YAP1* expression in 31 types of tumors, including ACC, BLCA, BRCA, CESC, CHOL, COAD, DLBC, ESCA, GBM, HNSC, KICH, KIRC, KIRP, LGG, LIHC, LUAD, LUSC, MESO, OV, PAAD, PRAD, READ, SARC, SKCM, STAD, TGCT, THCA, THYM, UCEC, UCS, UVM (Figure 6). Consistent with almost all results analyzed through TIMER database, there were strong positive correlations between the expression of *YAP1* and *SKP2* in ACC, BLCA, BRCA, CESC, COAD, DLBC, ESCA, GBM, HNSC, KICH, KIRC, KIRP, LGG, LIHC, LUAD, LUSC, OV, PAAD, PRAD, SARC, SKCM, STAD, TGCT, THCA, UCEC, UCS, UVM through GEPIA database (Supplementary Figure S2). But *CDC20* expression exhibited an inconsistent trend with *YAP1* expression. *CDC20* expression was positively correlated with *YAP1* expression in ACC, PAAD, PCPG, TGCT, and UCEC, whereas negatively correlated with *YAP1* expression in BRCA, COAD, LUAD, READ, and THYM (Supplementary Figure S3). And there was no significant relationship between *CDC20* and *YAP1* expression in other tumors. Hence, *SKP2*, instead of *CDC20,* positively correlates with *YAP1* expression in pan-cancer.

### *SKP2,* as the Target of YAP, Promotes Cell Proliferation in the Cell Cycle Process of Pan-Cancer

**FIGURE 7:**
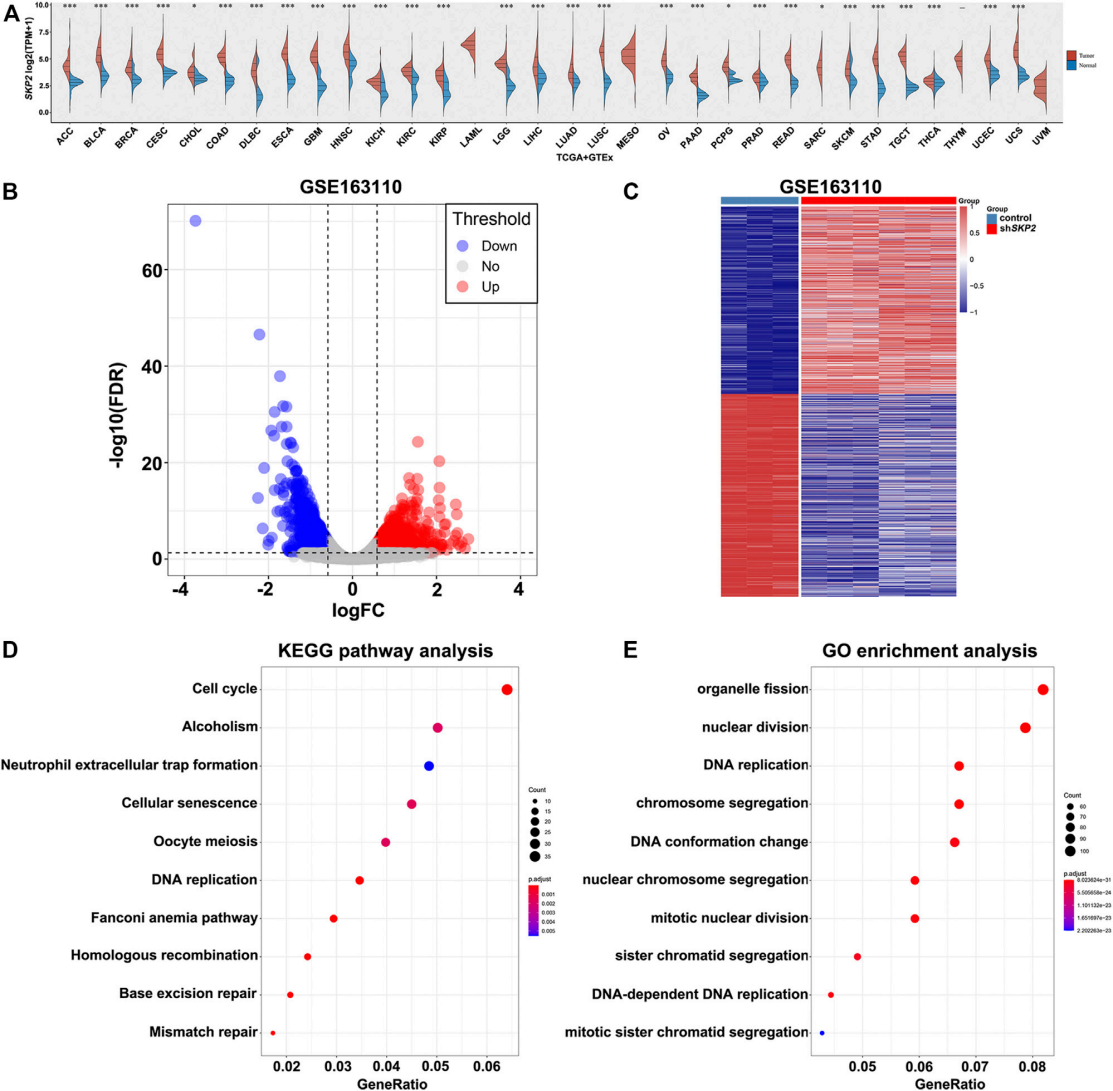
The expression analysis of *SKP2* in pan-cancer and its functional analysis. **(A)** The expression level of *SKP2* in different cancer types from TCGA and GTEx data. **(B)** Volcano map for DEGs between control and sh*SKP2* group with *p* value< 0.05, **|**Fold Change**|** >1.5 as the cut-off criteria. **(C)** Heat map for hierarchical clustering of DEGs. **(D)** The top 10 KEGG pathways of DEGs. **(E)** The significant-top 10 GO functional annotations.

To better understand the role of Skp2 played in pan-cancer, we firstly compared the *SKP2* expression using the data from TCGA and GTEx databases. Interestingly, the upregulated *SKP2* mRNA expression was observed consistently in tumor tissues versus normal tissues in 29 types of human common cancer, including ACC, BLCA, BRCA, CESC, CHOL, COAD, DLBC, ESCA, GBM, HNSC, KICH, KIRC, KIRP, LGG, LIHC, LUAD, LUSC, OV, PAAD, PCPG, PRAD, READ, SARC, SKCM, STAD, TGCT, THCA, UCEC, and UCS (Figure 7A). The remaining four types of cancer, including LAML, MESO, THYM, and UVM, lack the expression data of *SKP2* in normal tissue (Figure 7A). These findings revealed that Skp2 acted as an oncogenic protein in multiple cancers.

To further investigate the molecular function of Skp2 in tumors, the analysis of differential expression of mRNAs was performed between the control and sh*SKP2* group using the Limma package of R software in GSE163110. A total of 1,520 DEGs were observed in the control group compared to the sh*SKP2* group with **|**Fold Change**|** >1.5 and *p* < 0.05 as the cut-off criteria. Volcano and heat maps of these genes were drawn as shown in Figures 7B,C.

In order to determine the functions and pathways of DEGs related to the inhibition of *SKP2*, we performed the KEGG pathway and GO analysis. The top 10 KEGG pathways of the DEGs indicated that most DEGs were significantly enriched in the cell cycle signaling pathway (Figure 7D). And the 10 most significant GO functional annotations were enriched in the nuclear division, DNA replication, and so on (Figure 7E). These analysis results above indicated that Skp2 played an extremely important role in nuclear division and DNA replication in the cell cycle.

**FIGURE 8:**
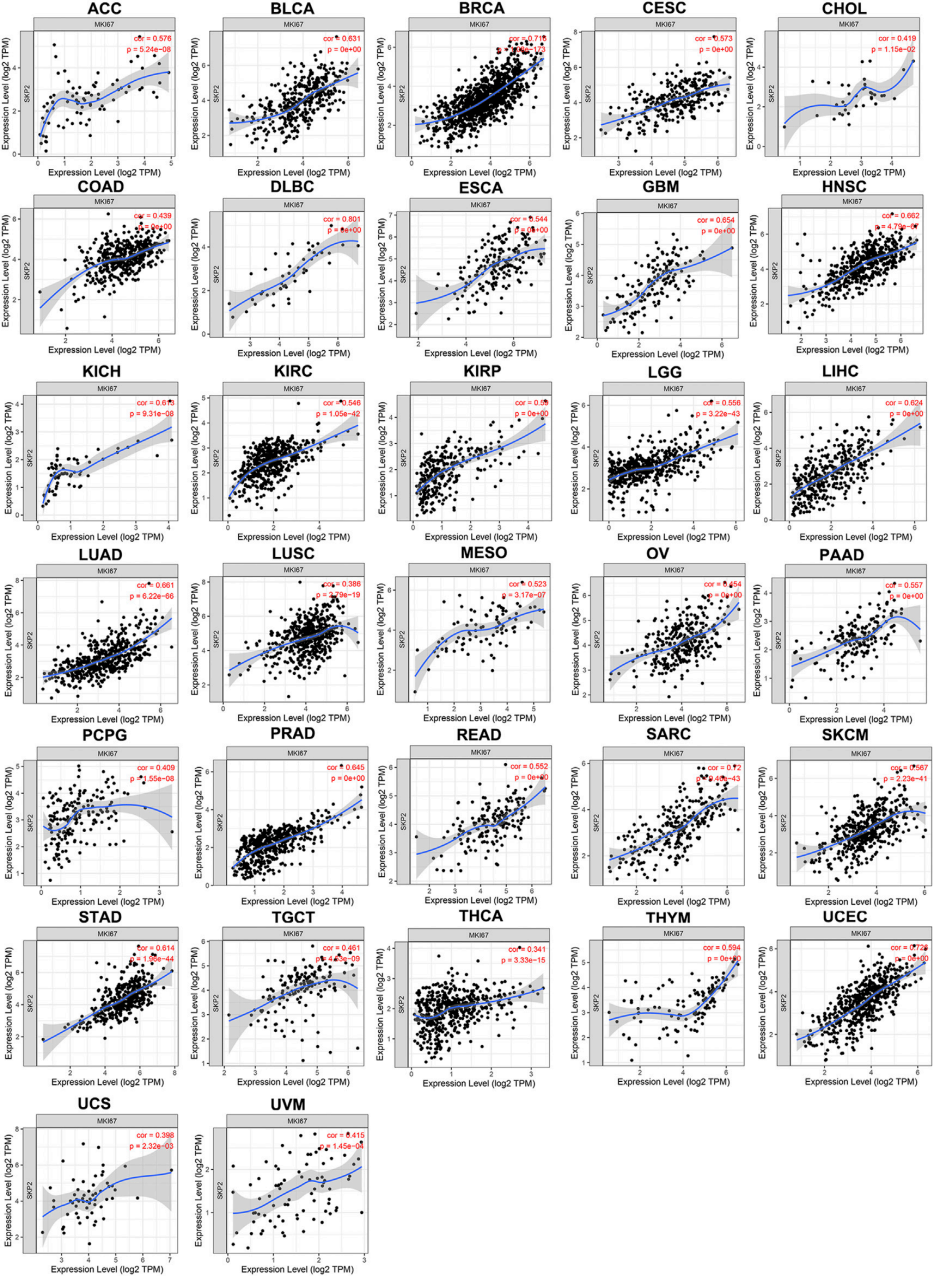
The positive correlation between *SKP2* and *MKI67* expression analyzed by TIMER in pan-cancer.

As Ki67 was a vital important proliferation indicator in the cell cycle, we assessed the relationship between *SKP2* and *MKI67* mRNA in pan-cancer using TIMER database. TIMER results noted that *SKP2* expression was positively correlated with *MKI67* expression in all types of tumors included in TIMER database (Figure 8). Taken together, *SKP2,* as the target of YAP, promotes cell proliferation in the cell cycle process of pan-cancer.

### *SKP2* Expression Negatively Correlates With MLN4924 IC50 Values and Its Overexpression Increases Cancer Cell Sensitivity Towards MLN4924 in Pan-Cancer

**FIGURE 9:**
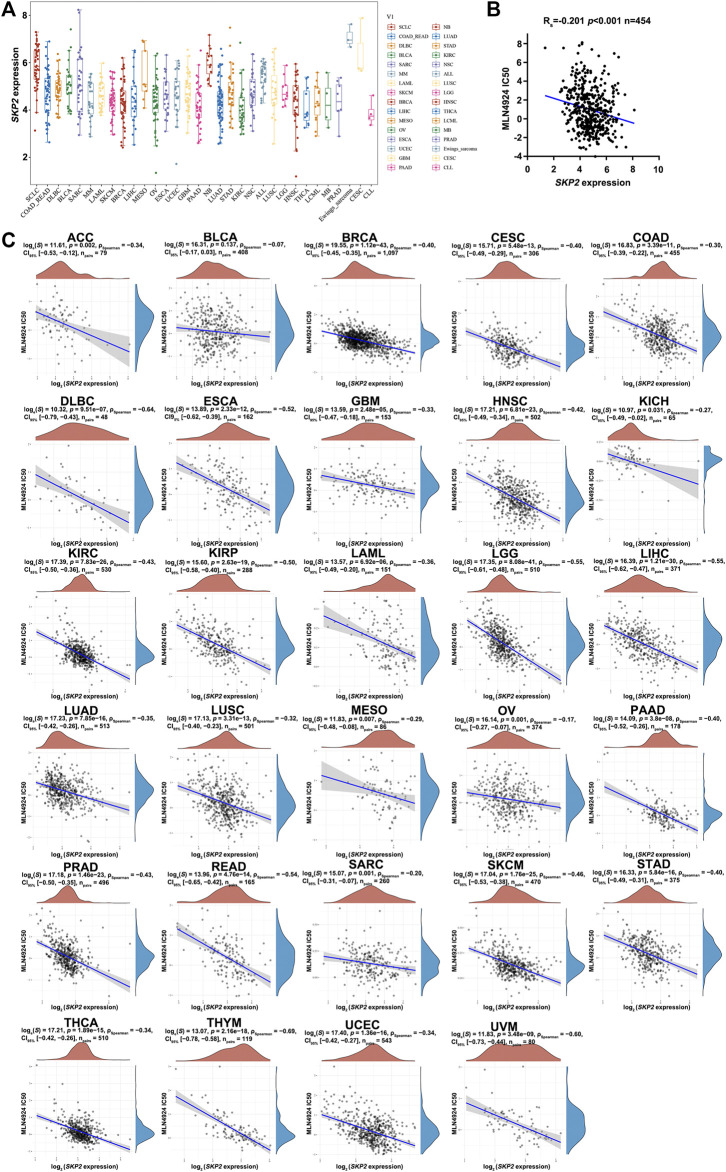
The negative correlation between *SKP2* expression and MLN4924 IC50 score in pan-cancer **(A)** The expression distribution of *SKP2* in different tumor tissues, where the horizontal axis represents samples from different groups, and the vertical axis represents the expression distribution of *SKP2*. **(B)** MLN4924 IC50 values of 454 human cancer cell lines from the CCLE dataset were negatively correlated with *SKP2* expression from the TCGA database. Spearman r and statistics are indicated. **(C)** Spearman correlation analysis of MLN4924 IC50 score and *SKP2* mRNA expression in pan-cancer.

MLN4924 is an agent that targets the NEDD8-activating enzyme, thus affecting CUL1 neddylation and impairing Skp2-SCF complex formation and ubiquitin ligase activity. It was previously evaluated by the Pediatric Preclinical Testing Program, and it exhibited effective activity *in vitro* and could inhibit tumor growth (Chen and Tweddle, 2012). Another previous study reported that the activity of Skp2 could be inhibited by MLN4924 and subsequently triggered an apparent stabilization of p27, which in turn induced cell cycle arrest in PC3 cells (Mickova et al., 2021). To demonstrate the functional relevance of Skp2 in modulating MLN4924 sensitivity, we firstly assessed the expression of *SKP2* in pan-cancer cell lines from the CCLE dataset (Figure 9A). Further, we explored the association between *SKP2* expression and MLN4924 IC50 values in human cancer cell lines and pan-cancer. As shown in Figures 9B,C, the expression of *SKP2* was negatively correlated with MLN4924 IC50 values in 454 human cancer cell lines (Figure 9B) and in almost all cancer types, including ACC, BLCA, BRCA, CESC, COAD, DLBC, ESCA, GBM, HNSC, KICH, KIRC, KIRP, LAML, LGG, LIHC, LUAD, LUSC, MESO, OV, PAAD, PRAD, READ, SARC, SKCM, STAD, THCA, UCEC, and UVM (Figure 9C). Besides, we detected the mRNA level and protein expression of *SKP2* in various cancer cell lines in several cancer types. As shown in Supplementary Figures S4A,B, the expression of *SKP2* in MiaPaCa-2 was higher than that of BxPC-3, and the expression of *SKP2* in LN229 was higher than that of U87. Then we performed CCK8 assay on these cells, which were treated with different concentrations of MLN4924. Similar to Skp2 knockdown, BxPC-3 was less sensitive to MLN4924 than MiaPaCa-2, with an IC50 of 213 nM (Supplementary Figure S4C). Similarly, U87 was less sensitive to MLN4924 than LN229, with an IC50 of 4.28 μM (Supplementary Figure S4D). All data above suggested that the elevation of *SKP2* expression could increase cancer cell sensitivity towards MLN4924 in pan-cancer.

## Discussion

The mechanism of cancer occurrence and development is complicated, and it is generally believed to be closely related to the dysfunction of cell signaling pathways. With the identification of the components of the Hippo pathway, it was proved that Hippo/YAP pathway could control the organ size by regulating the balance between mammalian cell proliferation and apoptosis (Johnson and Halder, 2014). Nowadays, studies indicated that YAP was pervasively activated in human malignancies and its activation could induce cancer stemness, proliferation, metastasis, and chemoresistance (Zanconato et al., 2016). In our integrative pan-cancer analysis, the results showed that overexpression of *YAP1* only predicted poor prognosis in PAAD and LGG, which was inconsistent with the reported that YAP overexpression predicted worse OS in LIHC, ESCA, BLCA, and BRCA (Wu et al., 2019). The differences of results might major come from the difference of detecting methods. The data of the online datasets were from the results of RNA sequencing, while immunochemistry was used to detect the total YAP and nuclear YAP protein expression. And nuclear YAP was the active form of YAP. It was reported that large numbers of cancer-associated factors, including changes in mechanotransduction, oncogenic signaling, inflammation, and inhibition of the Hippo pathway, could conspire to promote the activation of YAP (Zanconato et al., 2016). YAP stands at the intersection of multiple signaling pathways and cooperates with other transcription factors to regulate the transcription of target genes, thereby contributing to the malignant progression of tumors (Toloczko et al., 2017; Totaro et al., 2017; Xu et al., 2021). Therefore, it was very important to elucidate the potential role of YAP in cancer development.

Pan-cancer analysis is essential for comparing the similarities and differences between different cancers and is very helpful to provide new insights into cancer treatment. In this study, the *YAP1* expression pattern and its prognostic value in patients with cancer were analyzed. The analysis results further supported its oncogenic role in tumor progression. Previous studies reported that YAP could promote cell proliferation and inhibit cell differentiation in medulloblastomas (Fernandez-L et al., 2009). In view of these, we assessed the association between *YAP1* expression and tumor cell proliferation in pan-cancer. Intriguingly, we found that the expression of *YAP1* was positively correlated with the expression of *MKI67* in most of the cancer types. The results we presented here clearly indicated that YAP played an essential role in tumor cell proliferation across cancers.

Skp2 was an F-box protein of the SCF E3 ubiquitin-protein ligase complex (Méndez et al., 2002; von der Lehr et al., 2003). It was initially identified as a component of the cyclin-A-CDK2 complex and subsequently was proved to promote cell entry into S phase (Zhang et al., 1995). Since then, it has been widely characterized as an SCF ubiquitin ligase, which is essential for cell cycle progression and cell proliferation (Frescas and Pagano, 2008). For example, Skp2 is crucial for p27 and p21 degradation thereby limiting cells in the G1 phase, prior to entry into S-phase (Yu et al., 1998; Carrano et al., 1999; Tsvetkov et al., 1999; Chen et al., 2021). In our study, *SKP2* was validated as the downstream target of YAP involved in cell cycle progression in cancer, which was consistent with previous research that YAP directly regulates *SKP2* transcription (Stein et al., 2015; Jang et al., 2017). Here we found elevated *SKP2* expression was consistent in tumor tissues versus normal tissues in 29 types of human common cancer in pan-cancer analysis. And our analysis results indicated inhibition of *SKP2* hindered the progression of the cell cycle. Further, we assessed the correlation between *SKP2* and *MKI67* in pan-cancer. Strikingly, *SKP2* was positively correlated with the expression of *MKI67* in almost all cancer types. The evidence above was consistent with other reports that expression of Skp2 increased with androgen addition in human prostate cells, which subsequently resulted in increased degradation of p27 and promoted prostate cell proliferation (Waltregny et al., 2001; Lu et al., 2002). Collectively, these results indicated that the overexpression of Skp2, as the target of YAP, promotes the growth of cancer cells.

Of the ubiquitin ligases executing the cell cycle phases and checkpoints, Skp2-SCF complex has arguably the strongest biological rationale as a drug discovery target (Cardozo and Pagano, 2007). MLN4924, a protein neddylation inhibitor, could impair the complex formation and ubiquitin ligase activity (Chen and Tweddle, 2012). In preclinical studies, MLN4924 exhibited effective cytotoxicity against a group of human cancer cell lines and inhibited tumor growth in mouse xenograft models (Soucy et al., 2009a; Lin et al., 2010). For its potent antitumor activity and well-tolerated toxicity in preclinical studies (Soucy et al., 2009b; Milhollen et al., 2010; Swords et al., 2010), MLN4924 has entered a series of phase I/II/III clinical trials for patients with solid tumors or hematological malignancies. Currently, 41 clinical trials are enrolling patients in clinicaltrials.gov website, and several completed phase I clinical trials have demonstrated that MLN4924 is safe and feasible. Intriguingly, our study showed that *SKP2* expression negatively correlated with MLN4924 IC50 values in almost all cancer types. It suggested that high *SKP2* expression may increase the responsiveness towards MLN4924 in multiple cancer types. This was consistent with the reports that Skp2 drives the sensitivity to MLN4924 in malignant pleural mesothelioma (Salaroglio et al., 2022) and the sensitivity of RB cells to MLN4924 matches their dependency on Skp2 (Aubry et al., 2020). What’s more, it was reported that MLN4924 could inhibit the ubiquitination of Lats1 and 2 mediated by CRL4^DCAF1^, thereby inducing the phosphorylation and inactivation of YAP which led to the inhibition of proliferation in malignant pleural mesothelioma (Cooper et al., 2017). Therefore, MLN4924 inhibited the proliferation of tumor cells by blocking the ubiquitin ligase activity of Skp2 on the one hand, and downregulating *SKP2* expression by inducing the phosphorylation and inactivation of YAP on the other hand. To sum up, MLN4924 should be selected as a very appropriate drug for the treatment of cancers that exhibit overexpression of Skp2.

## Conclusion

Taken together, we presented meaningful findings with respect to YAP clinical significance in pan-cancer. Our analyses highlighted the potential role of YAP in the cancer cell proliferation of various cancers and revealed that Skp2 as the target of YAP promoted tumor cell proliferation in pan-cancer. Moreover, we provided evidence that higher *SKP2* expression increases the sensitivity of cancer cell lines towards MLN4924. Of course, more clinical and basic studies are required to further validate the findings presented herein. Thus, our results suggest MLN4924 is an attractive therapeutic drug for the treatment of YAP-driven cancers.

## Data Availability

The datasets presented in this study can be found in online repositories. The names of the repository/repositories and accession number(s) can be found in the article/Supplementary Material.
