# Supplementary material for: An Integrative Pan-Cancer Analysis Revealing MLN4924 (Pevonedistat) as a Potential Therapeutic Agent Targeting Skp2 in YAP-Driven Cancers
Source: Front Genet. 2022 May 24;13:866702. doi: 10.3389/fgene.2022.866702 (PMC9171011; doi:10.3389/fgene.2022.866702)
Supplement: Supplementary file 1 [file DataSheet2.docx]

**Supplemental Materials and Methods**

**Cell culture**

MiaPaCa-2, LN229, and SKOV3 were cultured in DMEM supplemented with 10% fetal bovine serum (FBS) in a 5% CO2 incubator at 37 °C. HCT116 (ATCC) was cultured in McCoy’s 5A medium supplemented with 10% FBS. Cells were tested and authenticated in Beijing Microread Genetics Co., Ltd. (Beijing, China) by short tandem repeat profiling. All cell lines were tested negative for mycoplasma contamination.

**Plasmid construction and transfection**

YAP specific shRNA (#1: GATCCgacatcttctggtcagagaCTCGAGtctctgaccagaagatgtcTTTTTG, #2: GATCCgcatcttcgacagtcttctttCTCGAGaaagaagactgtcgaagatgcTTTTTG), and scrambled sequence (GATCCgttctccgaacgtgtcacgtCTCGAGacgtgacacgttcggagaacTTTTTG) were synthesized and cloned into pLVX-shRNA2-Neo lentiviral vector, respectively. The plasmids were next transfected into HEK-293T cells with the packing plasmids pMD2G and psPAX2 to produce lentivirus. Stable lentivirus-infected cells were selected with G418 and verified by western blot analysis.

**Western blot analysis**

In brief, cells were lysed in ice-cold lysis buffer. Equal amounts of protein were loaded and separated by SDS-PAGE and then were transferred onto nitrocellulose membranes (Millipore, Billerica, MA, USA). The membranes were incubated overnight at 4℃ with the primary antibodies and were then treated with secondary antibodies (IRDye®800CW). Infrared signals were examined by using the Odyssey imaging system (Li-Cor Biosciences, Lincoln, NE, USA).

**Correlation analysis through GEPIA database**

This function was used to conduct the correlation analysis between *YAP1* and *SKP2* for any given sets of TCGA tumor expression data using Spearman correlation statistics.

**MLN4924 preparation for in vitro and IC50 determination**

MLN4924 was synthesized by AbMole, Inc. and was dissolved in DMSO to make a 1 mM stock solution and kept in −20 ℃ before use. Cell suspensions were seeded at 3,000~4,000 cells per well in 96-well plates in triplicates and treated with MLN4924 in various concentrations for 2 days or more. Cell viability was measured with Cell Counting Kit-8 (Sigma-Aldrich) according to the manufacturer's introductions. The optical density values were measured at 450 nm using a microplate reader.
