# Supplementary material for: An Integrative Pan-Cancer Analysis Revealing MLN4924 (Pevonedistat) as a Potential Therapeutic Agent Targeting Skp2 in YAP-Driven Cancers
Source: Front Genet. 2022 May 24;13:866702. doi: 10.3389/fgene.2022.866702 (PMC9171011; doi:10.3389/fgene.2022.866702)
Supplement: Supplementary file 2 [file DataSheet1.docx]

**Supplemental Figures**

**Supplemental Figure S1. YAP was an upstream regulator that positively regulated the expression of Skp2, related to Figure 5.**

**
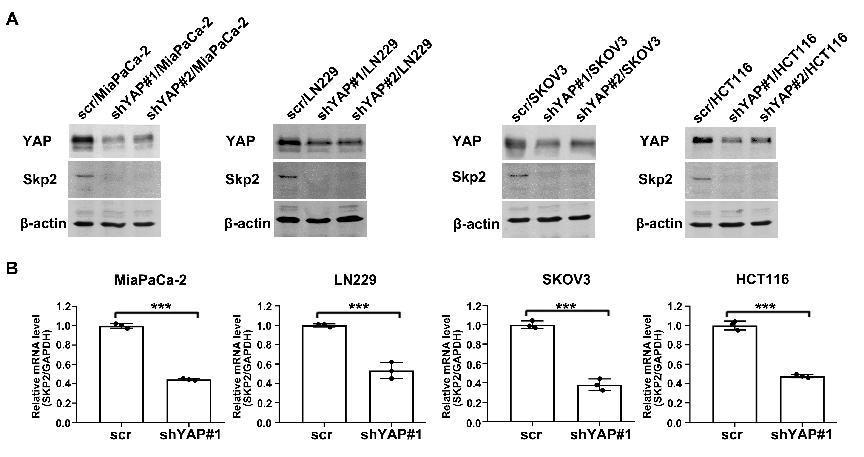
**

YAP was an upstream regulator that positively regulated the expression of Skp2. (A) Establishment and verification of stable cell lines underexpressing YAP. Representative samples of YAP and Skp2 in MiaPaCa-2, LN229, SKOV3, and HCT116 cell lines detected by western blotting. β-actin was used as an internal control. (B) Relative mRNA level of *SKP2* in MiaPaCa-2, LN229, SKOV3, and HCT116 cell lines with knockdown of YAP or not. Histogram (columns: mean, bars: standard deviation, n = 3), *p* values were calculated by Student’s *t*-test, ****p* < 0.001.

**Supplemental Figure S2. The positive correlation between *YAP1* and *SKP2* expression analyzed by GEPIA in pan-cancer, related to Figure 6.**


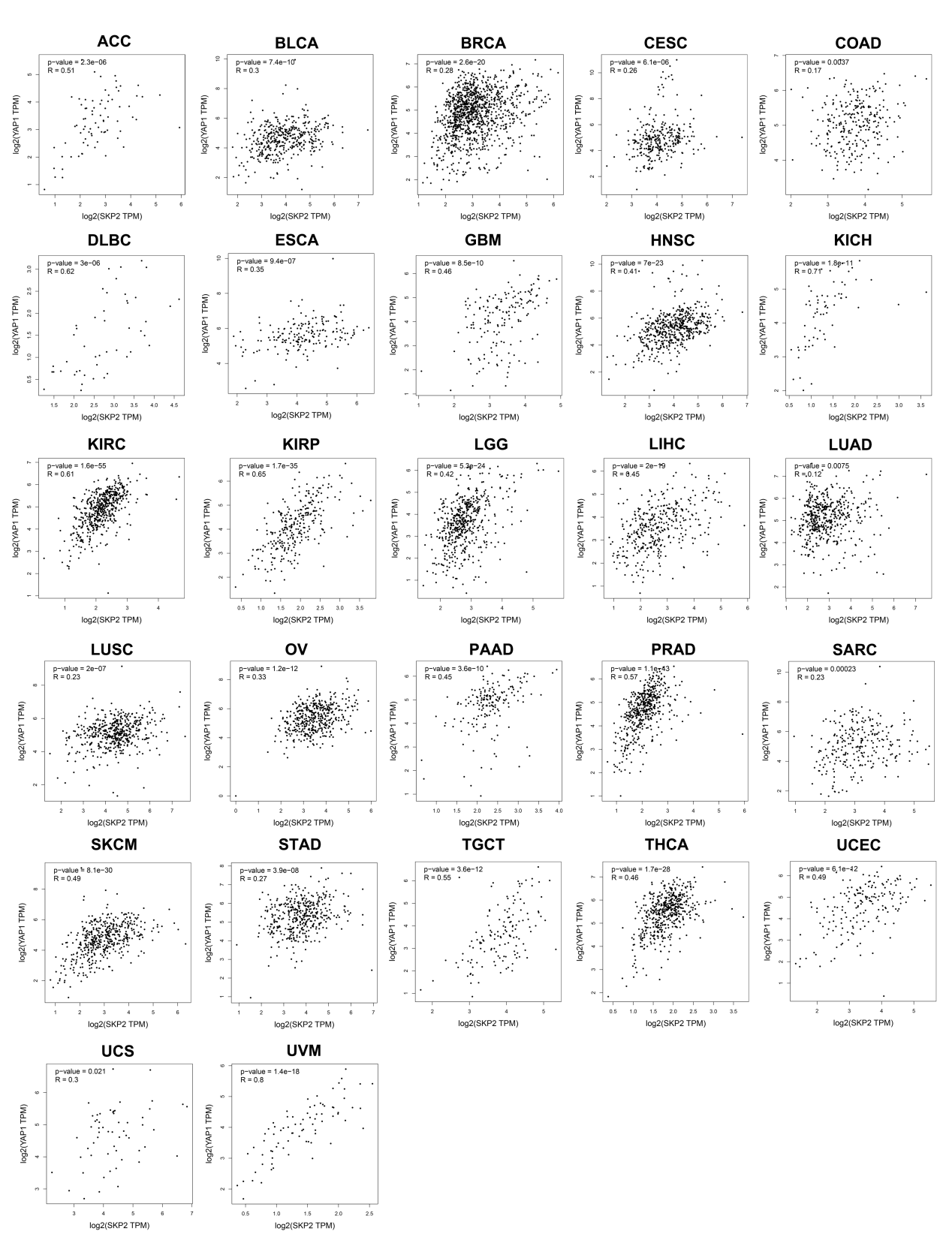


The positive correlation between *YAP1* and *SKP2* expression analyzed by GEPIA in pan-cancer

**Supplemental Figure S3. The inconsistent correlation between *YAP1* and *CDC20* expression analyzed by TIMER in pan-cancer.**


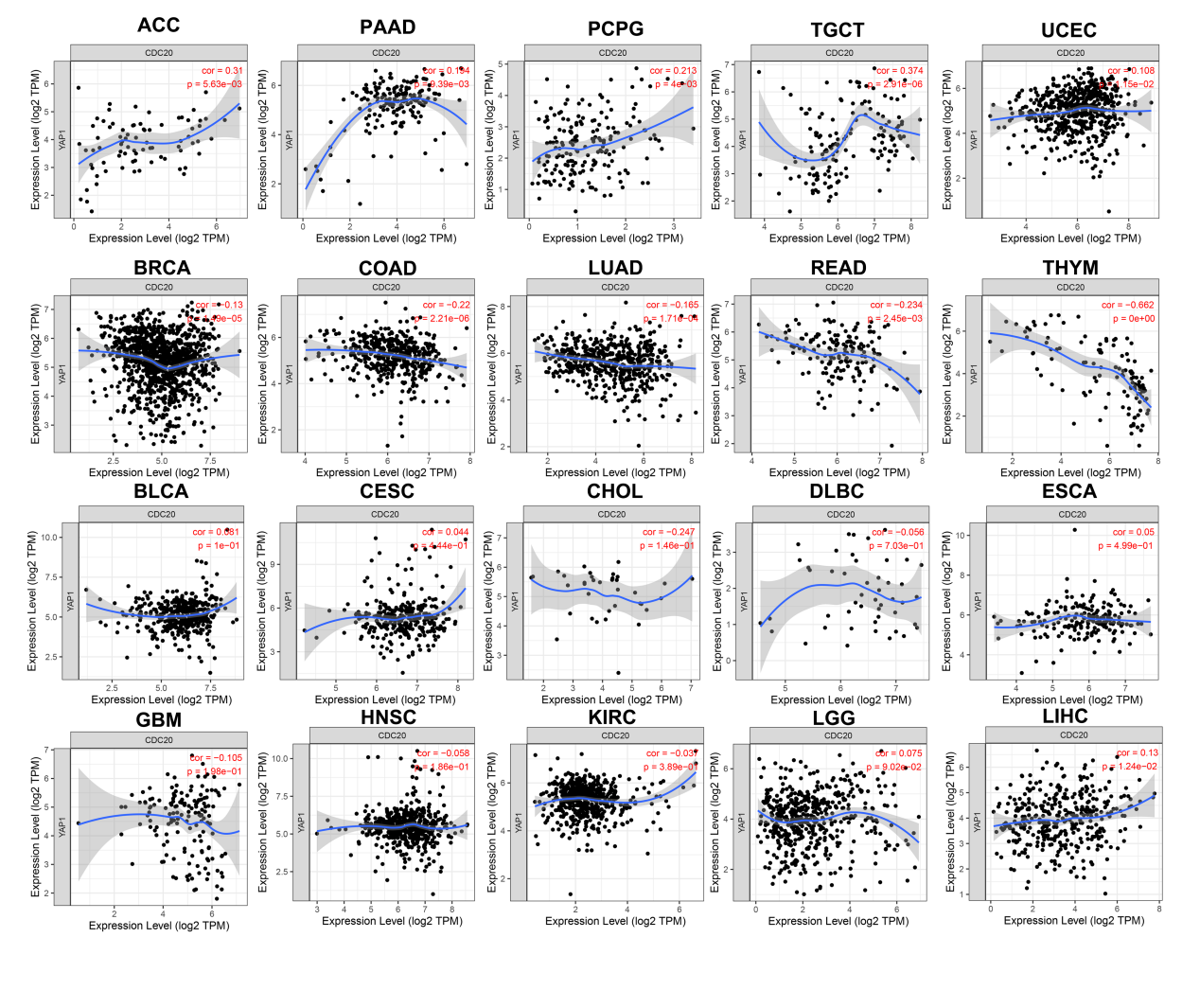


The inconsistent correlation between *YAP1* and *CDC20* expression analyzed by TIMER in pan-cancer.

**Supplemental Figure S4. Cancer cells highly expressing Skp2 were more sensitive to MLN4924, related to Figure 9.**

**
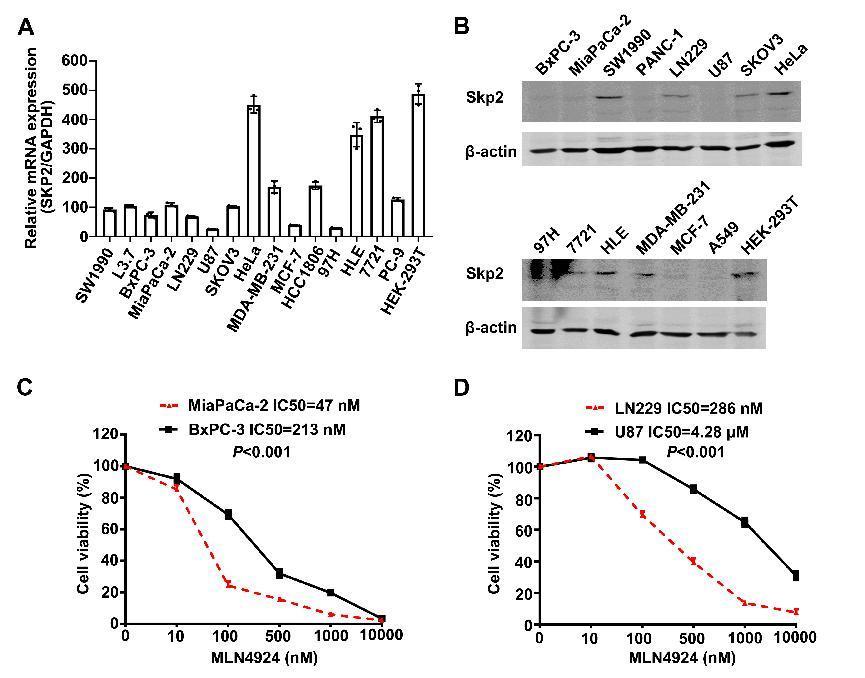
**

Cancer cells highly expressing Skp2 were more sensitive to MLN4924. (A) The mRNA level of *SKP2* in different cancer cell lines measured by RT-qPCR. (B) The expression level of Skp2 in different cancer cell lines measured by western blot. (C) The inhibitory dose-response curves for MLN4924 in MiaPaCa-2 and BxPC-3 cell lines. (D) The inhibitory dose-response curves for MLN4924 in LN229 and U87 cell lines.

**Supplemental Tables**

| **Supplemental Table 1. Correlation of YAP expression to clinicopathological features in PAAD.** | | | | |
| --- | --- | --- | --- | --- |
| **Parameters** | **YAP** | | **χ2** | P value |
|  | **Low** | **High** |  |  |
| **Age (years)** |  |  | **0.033** | **0.706** |
| **<60** | **19** | **50** |  |  |
| **≥60** | **15** | **46** |  |  |
| **Gender** |  |  | **-0.055** | **0.533** |
| **Male** | **17** | **54** |  |  |
| **Female** | **17** | **42** |  |  |
| **Histological grade** |  |  | **0.470** | 0.000a |
| **G1** | **17** | **8** |  |  |
| **G2** | **16** | **58** |  |  |
| **G3** | **1** | **30** |  |  |
| **Tumor size (cm)** |  |  | **-0.120** | **0.174** |
| **≤3** | **11** | **44** |  |  |
| **>3** | **23** | **52** |  |  |
| **pTNM stage** |  |  | **0.127** | **0.150** |
| **IB** | **13** | **21** |  |  |
| **IIA** | **15** | **55** |  |  |
| **IIB** | **6** | **20** |  |  |
| **LN metastasis** |  |  | **-0.009** | **0.921** |
| **negative** | **27** | **77** |  |  |
| **positive** | **7** | **19** |  |  |
| **Abbreviation: LN,lymph node.** a**Statistically significant (**P **< 0.05).** | | | | |

| **Supplemental Table 2. Univariate and multivariate Cox proportional hazards analysis of clinicopathological factors for overall survival and relapse free survival.** | | | | |
| --- | --- | --- | --- | --- |
| Variables | **Overall survival** | | **Relapse free survival** | |
|  | HR (95.0% CI) | *P* value | HR (95.0% CI) | *P* value |
| **Univariate analysis** |  |  |  |  |
| Age | 1.022 (0.664-1.574) | 0.922 | 0.809 (0.537-1.220) | 0.312 |
| Gender | 0.948 (0.615-1.460) | 0.808 | 0.861 (0.571-1.298) | 0.476 |
| Histological grade | 2.183 (1.504-3.169) | **0.000**a | 1.563 (1.117-2.188) | **0.009**a |
| Tumor size | 1.044 (0.672-1.621) | 0.849 | 1.074 (0.710-1.623) | 0.736 |
| pTNM stage | 1.160(0.847-1.588) | 0.356 | 1.187 (0.884-1.595) | 0.254 |
| LN metastasis | 1.013 (0.584-1.756) | 0.964 | 1.216 (0.745-1.985) | 0.433 |
| YAP expression | 6.240 (3.054-12.750) | **0.000**a | 3.039 (1.773-5.208) | **0.000**a |
| **Multivariate analysis** |  |  |  |  |
| Histological grade | 1.378 (0.896-2.120) | 0.145 | 1.142 (0.784-1.664) | 0.490 |
| YAP expression | 5.082 (2.364-10.926) | **0.000**a | 2.801 (1.559-5.035) | **0.001**a |
| NOTE: Multivariate Cox proportional hazards analysis used backward selection model. Abbreviations: HR, hazard ratio; CI, confidence interval; LN, lymph node. aStatistically significant (*P* < 0.05). | | | | |

**Supplemental Table 3. Antibodies and primers, related to supplemental Figures.**

| **Antibodies** | Manufacturers | Cat. Number |
| --- | --- | --- |
| β-actin | Santa Cruz Biotechnology | sc-47778 |
| YAP | Cell Signaling Technology | 14074 |
| Skp2 | Santa Cruz Biotechnology | sc-74477 |
|  |  |  |

| **Primers** | Sequences (forward/reverse) | Usage |
| --- | --- | --- |
| *SKP2* | 5′‐ATGCCCCAATCTTGTCCATCT‐3′ | RT-qPCR |
|  | 5′‐CACCGACTGAGTGATAGGTGT‐3′ |  |
